# Supplementary material for: Ballistic Thermal Transport at Sub‐10 nm Laser‐Induced Hot Spots in GaN Crystal
Source: Adv Sci (Weinh). 2022 Nov 17;10(2):2204777. doi: 10.1002/advs.202204777 (PMC9839872; doi:10.1002/advs.202204777)
Supplement: Supplementary file 1 — Supporting Information [file ADVS-10-2204777-s001.pdf]

ADVANCED SCIENCE
Open Access

# Supporting Information

for *Adv. Sci.*, DOI 10.1002/advs.202204777

Ballistic Thermal Transport at Sub-10 nm Laser-Induced Hot Spots in GaN Crystal

*Dezhao Huang, Qiangsheng Sun, Zeyu Liu, Shen Xu, Ronggui Yang and Yanan Yue**

# Supplementary Information for

## Ballistic Thermal Transport at Sub-10 nm Laser-Induced Hot Spots in GaN Crystal

Dezhao Huang[a, #], Qiangsheng Sun[a, #], Zeyu Liu[b], Shen Xu[c], Ronggui Yang[d], Yanan Yue[a, *]

a. School of Power and Mechanical Engineering, Wuhan University, Wuhan, Hubei, 430072, China
b. Department of Applied Physics, School of Physics and Electronics, Hunan University, Changsha, Hunan, 410082, China
c. School of Mechanical and Automotive Engineering, Shanghai University of Engineering Science, Shanghai, 201620, China
d. School of Energy and Power Engineering, Huazhong University of Science and Technology, Wuhan 430074, China

[#] D.H. and Q.S. contributed equally to this work.

*Corresponding authors: Yanan Yue, Email: yyue@whu.edu.cn

## S1. Additional Details of the Experimental Setup

The incident light is a 532 nm continuous wave laser (CW) to achieve steady-state heating/Raman excitation of the tip-sample system. A portable Raman probe was used for Raman scattering acquisition. A silicon AFM tip (ScanSens, CSG01 series model) was coated with a 20 nm-thick gold layer with a ~ 35 nm curvature radius (Figure 1c shows its scanning electron microscopy (SEM) image performed on a field emission MIRA3 TESCAN scanning electron microscope operating at 5 kV in the lens mode). During the experiment, the laser beam is reflected on a long-pass filter, passed through the Raman probe assemblies, and focused on the point of contact between the needle tip and the sample. In this work, the minimum spot size of the laser beam is about 60um obtained by the knife-edge method measurement, and the Raman scattering signal is collected by the same BAC102 Raman probe microscope and transmitted to the spectrometer with a wave number range from 0.45 to 3000 cm-1 and a resolution of 2.15 cm-1. In addition, the Raman probe assemblies is mounted on a bracket on a three-axis translation stage, making it feasible to precisely control the laser focus on the nanotip in a narrow area of space.

Since the minimum spot of the laser beam (approximately 60 μm in diameter) is larger than the 25 μm height of the nanotip, it was necessary to carefully adjust the laser to focus exactly at the apex of the tip, reducing the effect of additional irradiation of the non-tip contact area of the GaN substrate or the tip base on the experiment. In addition, since a 20 nm thick gold (Au) layer was coated on the silicon

AFM tip and the optical absorption depth of Au at the Raman spectrometer excitation wavelength was 13.7 nm, indicating that the Raman signal from the tip was negligible. It also ensures that the Raman signal comes from the GaN substrate under near-field optical heating and not from other materials.

In adjusting the laser focus position, the optical position of the tip under the laser is found by subtly moving the BAC102 Raman probe microscope. Among other things, since the Rayleigh scattering intensity is directly related to the irradiated area on the sample, which is used to determine the laser's focus position and the degree of focus. This is done by first focusing the spot on the cantilever, which is best focused when the Rayleigh scattering signal is strongest. Then the spot is moved in the direction of the cantilever until it reaches the base of the tip, while keeping it constant in the other two directions. The spot next moves from the tip base toward the apex of the tip and is judged by the Rayleigh scattering signal to be focused to the tip, with the final laser spot located where the Rayleigh scattering signal can just be detected. In this way, once the laser is focused on the tip of the tip, the AFM is controlled so that the GaN substrate moves upward towards the tip and contacts it.

## S2. Molecular Dynamics Simulations of the Tip-substrate Model

The simulation setup is illustrated in **Figure S1a**. A GaN surface (12nm by 12nm in the contact plane, and 3 nm thick) is used as the contacting surface and the gold tip is placed right above the surface. The morse potential is used to model the interaction between the gold atoms[1] and the Tersoff potential is used to model the GaN (wurtzite) substrate. After first relaxing the structures under isobaric–isothermal conditions

(NPT) at 1 bar and 300 K for 3 ns, we simulate the structures in the microcanonical (NVE) ensemble with fixed boundary condition. All MD simulations in this work are performed using the open source Large-scale Atomic/Molecular Massively Parallel Simulator (LAMMPS) [2]. In this simulation, the system firstly is relaxed in a NVT ensemble with the temperature maintained at 300 K using a Nose/Hoover temperature thermostat for 40 ps. And NVE ensemble is used for 50 ps to check the system temperature and the energy equilibrium. .Transient pump-probe method [3, 4] is used to investigate the interfacial thermal conductance between tip and substrate. In this approach, the system is initially placed in the Nose´–Hoover thermostat at 300 K for relaxation. Then the microcanonical ensemble is used to maintain the conservation of the total energy for 20ps. Once the system reached thermal equilibrium, a pulsed energy of 60 fs is applied to the tip. Immediately after the pulse, the temperature of the tip reached 528 K while that of the substrate remained at 300 K. After the temperature difference has been established, the thermal resistance can be calculated by the equation:

$$\frac{dE_{tip}}{dt} = A \cdot (T - T_{tip}) / R \tag{S1}$$

where $E_{\text{tip}}$ is the total energy of the tip, $A$ is the area and $T_{\text{tip}}$ and $T_{\text{sub}}$ are the temperatures of the tip and the substrate. $R$ is the value of the thermal resistance, and the interfacial thermal conductance can be expressed as $G = A/R$. Energy relaxation with time is fitted and the result is shown in the green line in **Figure S1b**. At the beginning portion of the energy curve, there is a small mismatch between the fitting curve and the calculated $E_t$. This is because that after a 60 fs ultrafast heating, the

kinetic and potential energies are in a non-equilibrium state and the calculated MD temperatures could not represent the real temperature. However, the observed fitting mismatch at the initial portion will not affect the overall fitting result since it only lasts for several picoseconds. The fitting profile soundly matches the energy outputs from MD simulation, which validates this approach for *G* extractions. In addition, the *G* value at this case is averaged from 5 independent simulations.

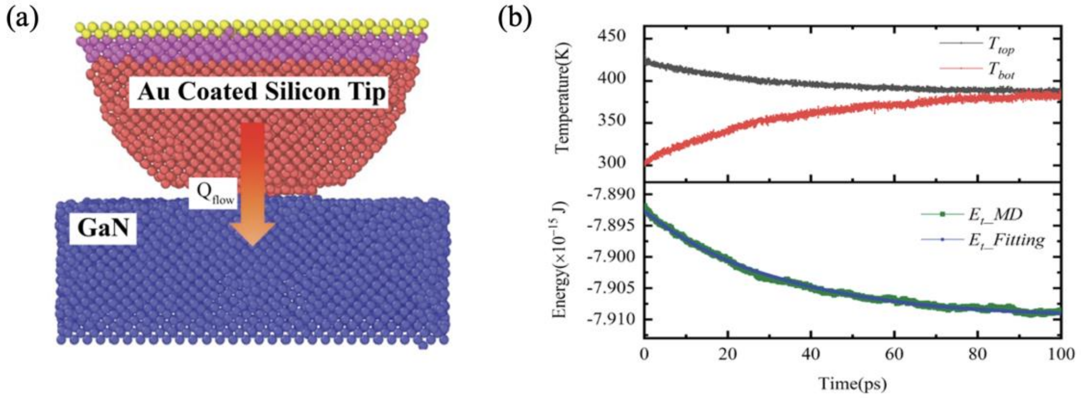


Figure S1. (a) An example tip-substrate model setup with an Au tip placed above the GaN substrate. Rigid layers are shown in yellow and thermostatted regions are shown in purple to conduct the interfacial thermal conductivity calculations. The size of the atoms is scaled according to their van der Waals radius. (b) Top panel represents the temperature of the top heated area and the bottom heat sink area as a function of the time. The lower panel depicts the energy time evolution.

## S3. Bulk GaN Sample Thickness

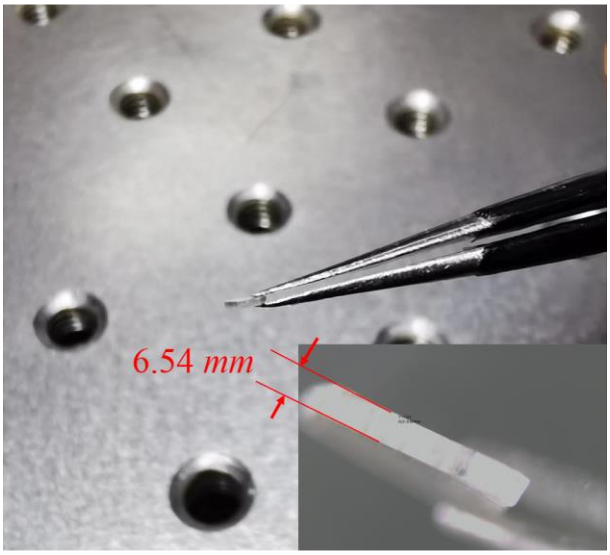


Figure S2. The GaN sample image displays the thickness of the GaN bulk sample.

## Reference:

[1] Kozlov ÉV, Popov LE, Starostenkov MD. Calculation of the morse potential for solid gold. Soviet Physics Journal, 1972, 15: 395-396

[2] Giles R. Parallel algorithms for short-range molecular dynamics. World Scientific Annual Review in Computational Physics, 1995, 3:

[3] Liu W, Wu Y, Hong Y, et al. Full-spectrum thermal analysis in twisted bilayer graphene. Phys Chem Chem Phys, 2021, 23: 19166-19172

[4] Zhang J, Hong Y, Tong Z, et al. Molecular dynamics study of interfacial thermal transport between silicene and substrates. Phys Chem Chem Phys, 2015, 17: 23704-23710
